# Effect of Particle Carriers for Intraperitoneal Drug Delivery on the Course of Ovarian Cancer and Its Immune Microenvironment in a Mouse Model

**DOI:** 10.3390/pharmaceutics14040687

**Published:** 2022-03-22

**Authors:** Roxanne Wouters, Sara Westrøm, Ann Vankerckhoven, Gitte Thirion, Jolien Ceusters, Sandra Claes, Dominique Schols, Tina B. Bønsdorff, Ignace Vergote, An Coosemans

**Affiliations:** 1 Laboratory of Tumor Immunology and Immunotherapy, Department of Oncology, Leuven Cancer Institute, KU Leuven, 3000 Leuven, Belgium; ann.vankerckhoven@kuleuven.be (A.V.); gitte.thirion@uzleuven.be (G.T.); jolien.ceusters@kuleuven.be (J.C.); an.coosemans@kuleuven.be (A.C.); 2 Oncoinvent AS, 0484 Oslo, Norway; westrom@oncoinvent.com (S.W.); bonsdorff@oncoinvent.com (T.B.B.); 3 Laboratory of Virology and Chemotherapy, Department of Microbiology, Immunology and Transplantation, Rega Institute, 3000 Leuven, Belgium; sandra.claes@kuleuven.be (S.C.); dominique.schols@kuleuven.be (D.S.); 4 Department of Obstetrics and Gynecology, Leuven Cancer Institute, University Hospitals Leuven, 3000 Leuven, Belgium; ignace.vergote@uzleuven.be; 5 Department of Oncology, Gynecological Oncology, KU Leuven, 3000 Leuven, Belgium

**Keywords:** calcium carbonate, PLGA, liposomes, drug carriers, microparticles, immune suppression, ovarian cancer

## Abstract

Novel treatment strategies are needed to provide a better prognosis for ovarian cancer. For this purpose, the current study was designed to evaluate the effects of different types of particle drug carriers on tumor response and on the tumor immune microenvironment (TME) after intraperitoneal (IP) administration in a murine tumor model. Mice with ID8-fLuc ovarian cancer were injected IP with pegylated liposomes, hydroxyapatite, polystyrene, poly(lactic-co-glycolic acid) (PLGA) and calcium carbonate (CaCO_3_) microparticles to evaluate the effect of the candidate carriers without drugs. Our results show that several types of microparticle drug carriers caused hyperproliferation of the tumor when injected IP, as reflected in a reduced survival or an accelerated onset of ascites. Alterations of the product formulation of CaCO_3_ microparticles could result in less hyperproliferation. The hyperproliferation caused by CaCO_3_ and PLGA was largely driven by a strong innate immune suppression. A combination with chemotherapy was not able to sufficiently counteract the tumor progression caused by the drug carriers. This research points towards the importance of evaluating a drug carrier before using it in a therapeutic setting, since drug carriers themselves can detrimentally influence tumor progression and immune status of the TME. However, it remains to be determined whether the hyperproliferation in this model will be of relevance in other cancer models or in humans.

## 1. Introduction

Ovarian cancer is the eighth leading cause of cancer-related deaths within the female population worldwide [1], with high-grade serous ovarian cancer (HGSOC, an epithelial subtype of ovarian cancer) being the most dominant subtype [2,3]. Patients often present with an already advanced disease stage (International Federation of Gynecology and Obstetrics (FIGO) stage III and IV) at the time of diagnosis due to the lack of symptoms at earlier stages of the disease. Standard treatment consists of radical cytoreductive debulking surgery combined with platin-based chemotherapy. About 70% of these patients will go in complete remission, but 80% of them eventually relapse. Overall, patients in an advanced disease stage have a poor five year survival of only 25% [4]. Targeted therapies for ovarian cancer include bevacizumab (anti-vascular endothelial growth factor receptor 2) and poly (ADP-ribose) polymerase (PARP) inhibitors. Immune checkpoint inhibitors are being investigated in clinical studies but have disappointing efficacy results so far [5,6].

Another type of targeted therapy that is currently investigated is the use of particle drug carriers designed to enhance specific drug retention within the peritoneal cavity in an attempt to increase local efficacy and reduce systemic toxicity of the drug under investigation [7,8]. A wide range of materials have been proposed as drug carriers: inorganic such as calcium carbonate (CaCO_3_) and hydroxyapatite; polymers such as poly (lactic-co-glycolic acid) (PLGA) and polystyrene; and lipid-based, where liposomes are the most well-known example. An important focus in the literature goes towards optimizing intraperitoneal (IP) delivery of chemotherapeutics [7,9]. In preclinical studies, all the particle drug carriers mentioned above have been shown to either enhance retention of a chemotherapeutic drug, prolong survival, reduce tumor volume or provide complete tumor regression in mice with ovarian cancer or other tumor types [10,11,12,13]. So far, the research has not translated into any particle drug carriers being approved for intraperitoneal use in the clinic, but two different nanoparticle-based systems for IP delivery of paclitaxel have been evaluated in phase I or II trials [14,15]. However, intravenous administration of pegylated liposomes carrying the chemotherapeutic doxorubicin is used as a second-line chemotherapy regimen for relapsed platinum-sensitive ovarian cancer patients in combination with carboplatin [16,17]. Additionally, the use of drug carriers in several non-chemotherapeutic applications holds promise for ovarian cancer. For example, CaCO_3_ microparticles were proven to be efficient drug carriers for local delivery of α-radionuclide therapy in preclinical models for ovarian cancer and have also been investigated in models of melanoma [18,19,20].

It is clear that drug carriers are of interest in the exploration of new therapeutic avenues. However, less attention is paid to evaluation of their influence on tumor behavior and interaction with the immune system, without the drug of investigation being present. Evidence is emerging that manipulations of the immune system can heavily impact the cancer course [21,22,23,24,25,26]. Therefore, the aim of the current research paper was to evaluate the effects that several commonly investigated drug carriers could have on tumor development in an immune-competent ovarian cancer mouse model and to assess their effects on both the adaptive and innate immune compartment of the tumor immune microenvironment (TME).

## 2. Materials and Methods

### 2.1. Ovarian Cancer Tumor Model

The ID8-fLuc cell line was transduced with a lentiviral vector (pCHMWS_CMV-fluc-I-PuroR) by the Laboratory of Molecular Virology and Gene Therapy and Leuven Viral Vector Core in our institute. Female C57BL/6 mice (Envigo, Horst, The Netherlands) of seven to nine weeks of age were inoculated intraperitoneally (IP) with 5 × 10^6^ ID8-fLuc ovarian cancer cells on day 0 of the experiment, in the lower right quadrant of the abdomen. All animal experiments were approved by the ethical committee (P123/2017) and followed the most recent ethical standards (NIH guidelines for the Care and Use of Laboratory Animals and EU Directive 2010/63/EU as amended by Regulation (EU) 2019/1010) and the ARRIVE (Animal Research: Reporting of In Vivo Experiments) guidelines [27,28].

### 2.2. Drug Carrier Administration in Mice

Polystyrene (Thermo Fisher Scientific, Waltham, MA, USA), PLGA (Phosphorex, Hopkinton, MA, USA) and hydroxyapatite (Plasma Biotal Ltd., Tideswell, UK) microparticles were dispersed in 0.9% NaCl (B. Braun, Melsungen, Germany) to obtain the required concentration. Pegylated liposomes (Avanti, Birmingham, AL, USA) were used as prepared by the manufacturer (dispersed in 10 mM histidine buffer with 10% *w*/*v* sucrose). The CaCO_3_ microparticles were prepared as described previously [18,19], and dispersion before autoclaving was performed in either 0.9% NaCl (CaCO_3_-MP-A), 0.9% NaCl and 2.4% (*w*/*w*) ethylenediamine tetra(methylene phosphonic acid) (CaCO_3_-MP-B) or 0.1 M Tris (CaCO_3_-MP-C). In addition, a non-autoclaved variant in 0.9% NaCl without additive (CaCO_3_-MP-D) was prepared. Particle diameters were approximately 5, 2, 3.5, 4 and 0.09 µm for CaCO_3_, polystyrene, PLGA, hydroxyapatite and pegylated liposomes, respectively, as described by the particle manufacturers. All microparticle preparations were made without addition of active drugs. Mass doses for all particle drug carriers ranged between 3 and 10 mg per mouse. All drug carriers were administered through IP injection.

### 2.3. Chemotherapy Treatment in Mice

Carboplatin (Hospira, ONCO-TAIN, Pfizer, New York, NY, USA) was dissolved in Dulbecco’s phosphate-buffered saline (DPBS, Thermo Fisher Scientific, Waltham, MA, USA) and administered IP at a dose of 80 mg/kg calculated for an average body weight of 20 g per mouse. Pegylated liposomal doxorubicin (Caelyx^®^, Janssens Cilag International NV, Beerse, Belgium) was administered IP at a dose of 1.6 mg/kg. Four out of 30 animals treated with the chemotherapy regimen experienced severe short-term toxicities (severe weight loss, diarrhea and cachexia) as a reaction to the chemotherapy in the first ten days after treatment administration and were sacrificed and excluded from all further data analysis.

### 2.4. Experimental Design

**Figure 1:**
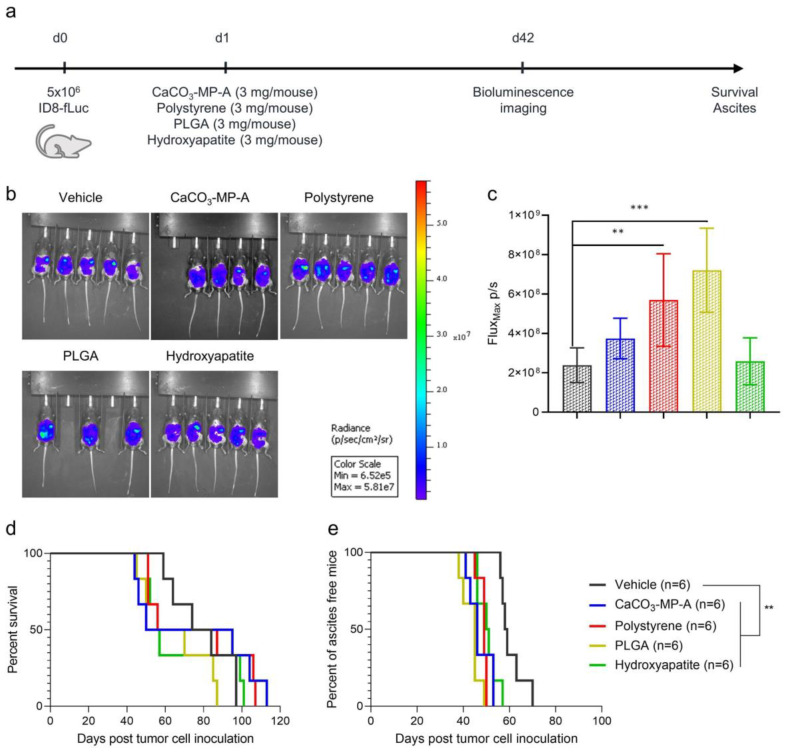
Experimental set-up of the experiment (**a**) with corresponding bioluminescence imaging visualization (**b**) and quantification (**c**) and Kaplan–Meier curves for survival and time to ascites development (**d**,**e**) of mice injected with vehicle control, 3 mg of CaCO_3_-MP-A, 3 mg of polystyrene, 3 mg of PLGA or 3 mg of hydroxyapatite. Mice showing signs of ascites development at the time point of bioluminescence imaging, were excluded from the analysis. (**b**,**c**): An increased tumor load was observed in mice injected with polystyrene and PLGA (*p*_adj_ = 0.0086 and *p*_adj_ = 0.0006, respectively). (**d**): No effects on survival were observed between the different groups. (**e**): Mice injected with CaCO_3_-MP-A, polystyrene, PLGA and hydroxyapatite showed a significant acceleration of malignant ascites development compared with vehicle control (*p*_adj_ = 0.0011, *p*_adj_ = 0.0011, *p*_adj_ = 0.001 and *p*_adj_ = 0.0033, respectively). PLGA: polymer poly (lactic-co-glycolic acid). Degree of significance: *p*_adj_ < 0.01 (**), *p*_adj_ < 0.001 (***).

**Figure 2:**
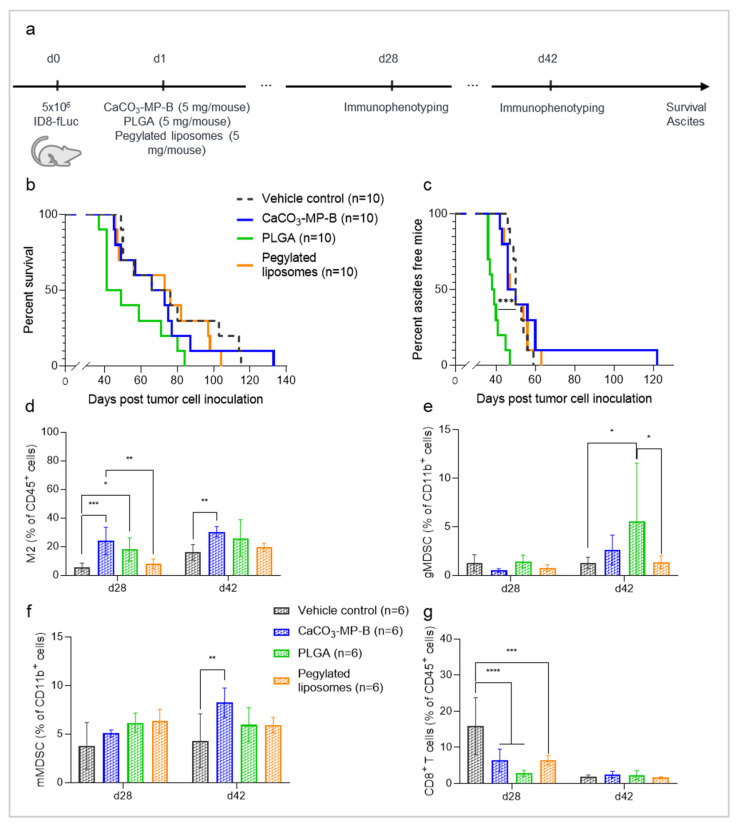
Experimental set-up of the experiment (**a**) with corresponding Kaplan–Meier curves for survival and time to ascites development (**b**,**c**) and immune profile of the tumor microenvironment on day 28 and 42 post tumor cell inoculation (**d**–**g**) of mice injected with vehicle control, 5 mg of CaCO_3_-MP-B, 5 mg PLGA or 5 mg pegylated liposomes. (**b**): No differences in survival were observed between the different groups. (**c**): Mice injected with PLGA did show a significant acceleration of ascites development compared with all other groups (*p*_adj_ = 0.0006). (**d**–**f**): In general, PLGA and CaCO_3_ elicit a strong immune suppressive tumor microenvironment reflected by increases in M2 macrophages and MDSC, while pegylated liposomes have a more favorable innate immune profile. (**g**): The CD8 + T cells were suppressed by all three drug carriers. PLGA: polymer poly (lactic-co-glycolic acid), PLD: pegylated liposomal doxorubicin, MDSC: myeloid-derived suppressor cell. Degree of significance: *p*_adj_<0.05 (*), *p*_adj_ < 0.01 (**), *p*_adj_ < 0.001 (***), *p*_adj_ < 0.0001 (****).

**Figure 3:**
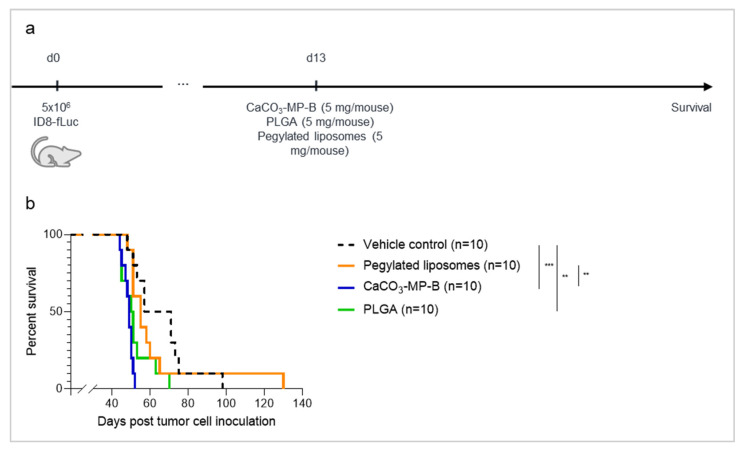
Experimental set-up of the experiment (**a**) with corresponding Kaplan–Meier survival curve (**b**) of mice injected with vehicle control, 5 mg of CaCO_3_-MP-B 5 mg PLGA or 5 mg pegylated liposomes on day 13 post tumor cell inoculation. The current data are obtained from the same experiment as the data that are found in Figure 6. (**b**): As a single treatment, CaCO_3_-MP-4 and PLGA have a shorter survival compared with vehicle control (*p*_adj_ = 0.0007 and *p*_adj_ = 0.0095, respectively). Pegylated liposomes show a significantly better survival than CaCO_3_-MP-4 (*p*_adj_ = 0.0017). PLGA: polymer poly (lactic-co-glycolic acid). Degree of significance: *p*_adj_ < 0.01 (**), *p*_adj_ < 0.001 (***).

**Figure 4:**
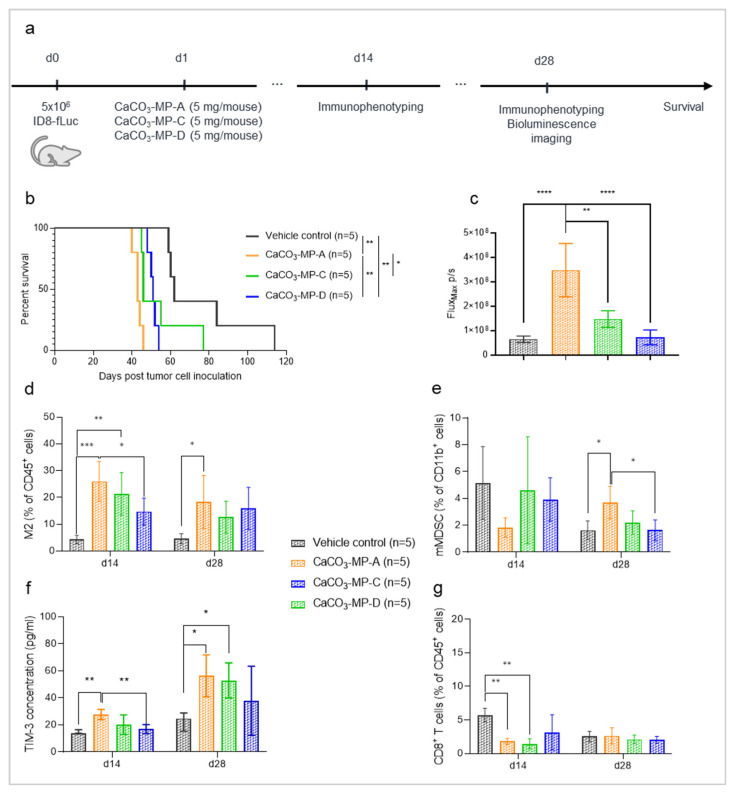
Experimental set-up of the experiment (**a**) with corresponding Kaplan–Meier survival curve (**b**), bioluminescence imaging quantification (**c**) and immune profile of the tumor microenvironment on day 14 and 28 post tumor cell inoculation (**d**–**g**) of mice injected with vehicle control, 5 mg of CaCO_3_-MP-C, 5 mg of CaCO_3_-MP-D or 5 mg of CaCO_3_-MP-A. (**b**,**c**): Different degrees of accelerated tumor progression were observed for all CaCO_3_ product formulations. (**d**–**f**): In general, a different degree of immune suppression in the tumor microenvironment is observed in response to CaCO_3_-MP-C, CaCO_3_-MP-D and CaCO_3_-MP-A, as reflected by the increase in M2 macrophages, mMDSC and the immune checkpoint TIM-3. (**g**): The CD8^+^ T cells were suppressed by all three product formulations of CaCO_3_. MDSC: myeloid-derived suppressor cell, TIM-3: T cell immunoglobulin and mucin domain-containing protein 3. Degree of significance: *p*_adj_<0.05 (*), *p*_adj_ < 0.01 (**), *p*_adj_ < 0.001 (***), *p*_adj_ < 0.0001 (****).

**Figure 5:**
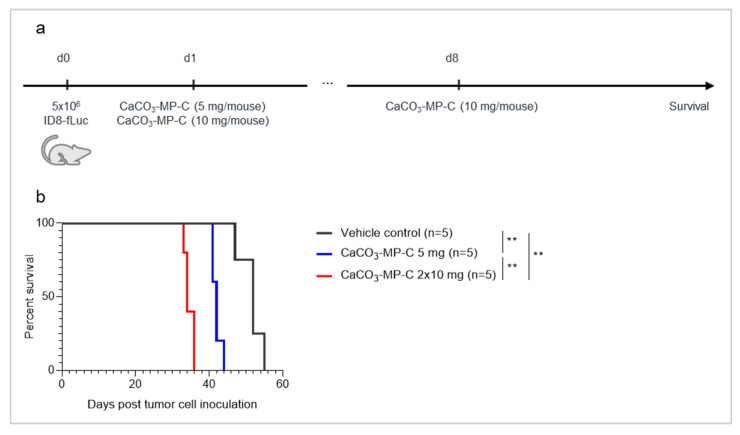
Experimental set-up of the experiment (**a**) with corresponding Kaplan–Meier survival curve (**b**) of mice injected with vehicle control, a single injection of 5 mg of CaCO_3_-MP-C on day 1 or a repeated injection of 10 mg of CaCO_3_-MP-C on day 1 and 8 post tumor cell inoculation. (**b**): A repeated dose of 2 × 10 mg of CaCO_3_-MP-C worsens survival compared with a single dose of 5 mg (*p*_adj_ = 0.0062), indicating a dose-dependent effect. Degree of significance: *p*_adj_ < 0.01 (**).

**Figure 6:**
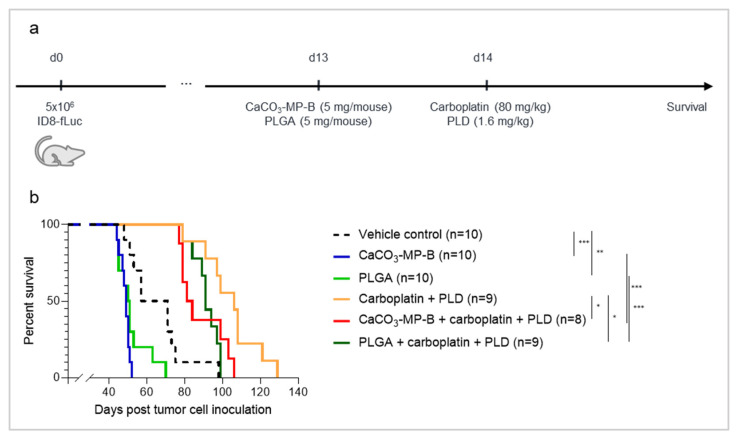
Experimental set-up of the experiment (**a**) with corresponding Kaplan–Meier survival curve (**b**) of mice injected with vehicle control, 5 mg of CaCO_3_-MP-B or 5 mg PLGA on day 13 and/or 80 mg/kg carboplatin with 1.6 mg/kg PLD on day 14 post tumor cell inoculation. Animals experiencing severe short-term toxicities (severe weight loss, diarrhea and cachexia) as a reaction to the chemotherapy in the first ten days after treatment administration, were excluded from all further data analysis. The current data are obtained from the same experiment as the data that are found in Figure 3. (**b**): As a single treatment, CaCO_3_-MP-B and PLGA have a shorter survival compared with vehicle control (*p*_adj_ = 0.0007 and *p*_adj_ = 0.0095, respectively). This hyperproliferation effect of CaCO_3_-MP-B and PLGA remains present in combination with carboplatin and PLD compared with carboplatin and PLD alone (*p*_adj_ = 0.0325 and *p*_adj_ = 0.0191, respectively). PLGA: polymer poly (lactic-co-glycolic acid), PLD: pegylated liposomal doxorubicin. Degree of significance: *p*_adj_<0.05 (*), *p*_adj_ < 0.01 (**), *p*_adj_ < 0.001 (***).

Different regimens of particle administration were explored. In experiments where mice were exposed to a single administration of CaCO_3_ in different product formulations, PLGA, polystyrene, hydroxyapatite or pegylated liposomes, this administration was performed on day 1 and day 13 post tumor cell inoculation (Figure 1a, Figure 2a, Figure 3a and Figure 4a). Day 1 post tumor cell inoculation was chosen as a time point to mimic minimal residual disease after a cytoreductive debulking surgery in patients, a situation highly relevant for future therapeutic strategies currently under development to target micro-metastatic disseminations in the peritoneal cavity. Additionally, mice were also exposed to a repeated administration of CaCO_3_ on day 1 and day 8 post tumor cell inoculation (Figure 5a). In the combination experiment, drug carrier administration with CaCO_3_ or PLGA on day 13 post tumor cell inoculation was combined with carboplatin and PLD treatment on day 14 post tumor cell inoculation in an attempt to mimic a situation where the effects of the particle drug carrier and chemotherapy would act simultaneously. In addition, pegylated liposomes as a single treatment on day 13 was evaluated. Different sub-analyses of this experiment were performed for the included monotherapy (Figure 3a) and combination groups (Figure 6a). An immune readout was performed on mice injected with different product formulations of CaCO_3_ on day 14 and 28 post tumor cell inoculation, and on mice injected with PLGA, CaCO_3_ and pegylated liposomes on day 28 and 42 post tumor cell inoculation (Figure 2a and Figure 4a). For all injection time points, control mice received either DPBS, 0.9% NaCl or Plasmalyte (Baxter, Deerfield, IL, USA) without additives as the appropriate vehicle control solution for their respective experimental treatment.

### 2.5. Bioluminescence Imaging

Mice were anesthetized using isoflurane in a 70:30 nitrous oxide/oxygen mixture at 2%. The mice were placed in a supine position in the IVIS Spectrum at the Molecular Small Animal Imaging Center facility of our institute. Mice showing signs of ascites development at the time point of imaging, were excluded from the scanning procedure since the presence of ascites strongly interferes with the photon flux measurement and will therefore cause an underestimation of tumor load.

### 2.6. Blood and Peritoneal Fluid Sampling for Immune Readout

Mice were anesthetized with 50 mg/kg pentobarbital (Dolethal, Vetoquinol, Magny-Vernois, France) via an IP injection prior to blood sampling using sodium heparinized microhematocrit capillary tubes. Whole blood was centrifuged at 800 RCF for 10 min. Serum was collected and stored at −80 °C for further cytokine quantification with Luminex^®^. Next, the animals were euthanized by cervical dislocation. Peritoneal washing with 10 mL of DPBS was performed to collect the circulating immune cells in ascites and from the peritoneal lining. Peritoneal washings were centrifuged for 5 min at 500 RCF and resuspended in DPBS. Supernatant was collected and stored at −80 °C for Luminex^®^ analysis. Using a Lymphoprep (Stemcell Technologies, Vancouver, Canada) gradient, immune cells were isolated from the cell suspension and resuspended in DPBS for flow cytometry.

### 2.7. Flow Cytometry

Dead cells were excluded by the use of eFluor780 fixable viability dye staining (Affymetrix Inc., San Diego, CA, USA). Subsequently, immune cells were stained for extracellular T cell and myeloid markers for which the antibody panels can be found in Supplementary Tables S1 and S2, respectively. Additionally, cells were stained for intracellular markers. For the T cell panel, the cells were permeabilized using the eBioscience Foxp3/Transcription Factor Staining Buffer Set (Thermo Fisher Scientific, Waltham, MA, USA) according to manufacturers’ protocol, before cells were stained for FoxP3. Permeabilization for the myeloid panel was performed using the Leucoperm (Bio-Rad Laboratories Inc., Kidlington, UK) for intracellular staining of CD206. Samples were subsequently acquired on the BD Canto II (BD Biosciences, San Jose, CA, USA). Analysis was performed using FlowJo Analysis software (TreeStar, Inc., Ashland, OR, USA).

### 2.8. Luminex^®^

T cell immunoglobulin and mucin domain-containing protein 3 (TIM-3) were measured with Luminex^®^, according to the manufacturers’ protocol using customized Procartaplex^TM^ Immunoassay Kits (Life Technologies, Merelbeke, Belgium). In short, 96-well plates were loaded with antigen-specific capture antibody-coated magnetic beads before samples; standards and blanks were added and incubated for two hours. Subsequently, biotinylated detection antibodies were added. The antibody/antigen complex was visualized using streptavidin-conjugated R-phycoerythrin. A magnetic plate washer was used for washing steps throughout the protocol. Read-out was performed with Bio-plex 200 system of Bio-Rad (Hercules, CA, USA). Concentrations of the proteins were determined using five parameter log curves generated by the Bio-Plex Manager 4.1.1 software (Bio-Rad, Hercules, CA, USA).

### 2.9. Statistical Analysis

A statistical power analysis was performed to determine sample sizes for all experiments. A power of at least 0.80 was reached with 5 to 10 mice per treatment group, depending on the type of experiment. Individual time to ascites development and survival curves were compared using the log-rank (Mantel–Cox) test. Adjustment for multiple comparisons was performed with the Benjamini and Hochberg procedure (with *Q* = 5%). Data from the immune readouts and bioluminescence imaging were tested for outliers using Grubbs’ method (*α* = 0.05). Normal distribution was assessed using a Shapiro–Wilk test (*n* < 8). If data were normally distributed, they were expressed as means and standard deviations; if data were not normally distributed, they were expressed as medians and interquartile ranges. For comparison between all the different groups, an ANOVA with Tukey’s multiple comparisons test or a non-parametric Kruskal–Wallis test with Dunn’s multiple comparisons test was used to compare normally and not normally distributed data, respectively. All data in this manuscript were normally distributed, unless stated otherwise. An (adjusted) *p* value < 0.05 was considered significant. The power analysis was performed in R version 3.5.1 (R Foundation for Statistical Computing, Vienna, Austria, https://www.R-project.org/, accessed on 18 March 2022); all other statistical analyses were performed using GraphPad Prism version 8.2.1 (GraphPad Software, San Diego, CA, USA).

## 3. Results

### 3.1. Different Types of Drug Carriers Worsen Disease Symptoms in an Ovarian Cancer Mouse Model

Four different drug carriers—namely, CaCO_3_, polystyrene, PLGA and hydroxyapatite—were evaluated for their effect on survival and onset of ascites symptoms. When administered at a dose of 3 mg per mouse, survival remained unaffected (Figure 1d). However, a significant acceleration of ascites development compared with vehicle control (median onset of ascites of 58.5 days) was observed for CaCO_3_-MP-A, polystyrene, PLGA and hydroxyapatite (median onset of ascites of 46, 49, 45 and 50.5 days with *p*_adj_ = 0.0011, *p*_adj_ = 0.0011, *p*_adj_ = 0.0011 and *p*_adj_ = 0.0033, respectively) (Figure 1e). Furthermore, an increased tumor load was confirmed through bioluminescence imaging for mice that received polystyrene and PLGA (*p*_adj_ = 0.0086 and *p*_adj_ = 0.0006, respectively) compared with vehicle control mice on day 42 post tumor cell inoculation (Figure 1b,c). No other relevant toxicities were observed after microparticle administration.

### 3.2. A Higher Microparticle Dose Can Result in a Hyperproliferation of the Tumor and an Increase in Innate Immune Suppression

The effects on survival and ascites development and the immune status of the tumor microenvironment were assessed in mice that received either 5 mg of PLGA, CaCO_3_ microparticles (CaCO_3_-MP-B) or pegylated liposomes on day 1 post tumor cell inoculation (Figure 2a). Mice injected with CaCO_3_ microparticles presented with an increase in M2 macrophages compared with vehicle control mice both at day 28 (*p*_adj_ = 0.0004) and at day 42 post tumor cell inoculation (*p*_adj_ = 0.0095), as well as an increase in the monocytic subtype of MDSCs (mMDSC) at the latter time point (*p*_adj_ = 0.0012) (Figure 2d,f). Additionally, also for PLGA, an increase in M2 macrophages compared with vehicle control was observed on day 28 post tumor cell inoculation (*p*_adj_ = 0.0212). However, in this case, at the latter analysis time point, an increase in the granulocytic subtype of MDSCs (gMDSC) was observed (*p*_adj_ = 0.0135) (Figure 2d,e). These results highlight that both of these particle drug carriers have a similar innate immune suppression profile, as reflected by the changes in M2 macrophages and MDSCs. However, they both affected a different subtype of MDSCs, which indicates a possible different mechanism for the immune response of CaCO_3_ and PLGA. In contrast, administration of pegylated liposomes did not induce the same innate immune suppression in the tumor microenvironment. In the group that received the pegylated liposomes, the fraction of M2 macrophages was significantly lower compared with the CaCO_3_-MP-B treatment group at the early analysis time point (*p*_adj_ = 0.0019) (Figure 2d). Furthermore, the presence of gMDSCs in this group at the latter analysis time point was reduced compared with PLGA (*p*_adj_ = 0.0158) (Figure 2e). A significant decrease in in CD8 + T cells compared with vehicle control was observed for all drug carriers included in the study: CaCO_3_-MP-B (*p*_adj_ < 0.0001), PLGA (*p*_adj_ < 0.0001) and pegylated liposomes (*p*_adj_ = 0.0001) (Figure 2g). There were no differences in the immune status of the tumors after treatment with pegylated liposomes and vehicle control, which can indicate a more favorable immune profile compared with the PLGA and CaCO_3_.

The differences in immune status between the drug carriers were not translated into any statistically significant effects on survival (Figure 2b), although the survival curves indicate a slight hyperproliferative effect after treatment with PLGA particles. This corresponds with the observed acceleration of ascites development for PLGA (median onset of ascites of 38.5 days) compared with both vehicle control (median onset of ascites of 50 days, *p*_adj_ = 0.0006) and the other particle types (CaCO_3_-MP-B: median onset of ascites of 48 days, *p*_adj_ = 0.0006, pegylated liposomes: median onset of ascites of 48.5 days, *p*_adj_ = 0.0006) (Figure 2c). In contrast, when the same drug carriers were administered on day 13 post tumor cell inoculation (Figure 3a), the survival was significantly shorter for both PLGA and CaCO_3_-MP-B compared with the control (Figure 3b), which suggests that the stage of tumor development is also a factor to consider. In addition, the survival of mice that received pegylated liposomes was significantly better than mice that received CaCO_3_-MP-B (Figure 3b) (median survival of 55 days compared with 49 days, respectively, with *p*_adj_ = 0.0017), which may further corroborate the more favorable immune profile of the liposomes.

### 3.3. Different Product Formulations of CaCO_3_-Microparticles Have Different Effects on Tumor Progression and Innate Immune Status

To assess whether differences could be observed between different product formulations of the same drug carrier, three CaCO_3_ microparticle formulations were evaluated for their effect on survival and the immune status of the TME, when administered at a dose of 5 mg (Figure 4a). Mice that received CaCO_3_ microparticles survived shorter than the vehicle control (median survival of 62 days), but the reduction was only statistically significant for the CaCO_3_-MP-A and CaCO_3_-MP-D product formulation (median survival of 43 and 51 days, respectively, with *p*_adj_ = 0.0018) (Figure 4b). Interestingly, mice that received CaCO_3_-MP-A showed a significantly worse survival compared with mice that received either CaCO_3_-MP-C or CaCO_3_-MP-D (*p*_adj_ = 0.0344 and *p*_adj_ = 0.0036, respectively). Additionally, for mice that received CaCO_3_-MP-A, the tumor load measured by bioluminescence imaging at day 28 post tumor cell inoculation was significantly higher compared with vehicle control (*p*_adj_ = 0.0001) and mice that received either CaCO_3_-MP-C (*p*_adj_ = 0.0015) or CaCO_3_-MP-D (*p*_adj_ = 0.0001) (Figure 4c).

In accordance with the negative influence on survival, the three CaCO_3_ microparticle formulations showed an increase in immune suppressive M2 macrophages at the earliest analysis time point (day 14 post tumor cell inoculation) (Figure 4d), although the increase only reached statistical significance for CaCO_3_-MP-A and CaCO_3_-MP-C (*p*_adj_ = 0.0002 and *p*_adj_ = 0.0021, respectively). At the latter analysis time point (day 28 post tumor cell inoculation), the trend of higher fraction of M2 macrophages compared with vehicle control remained but was only significant for the CaCO_3_-MP-A treatment group (*p*_adj_ = 0.0324), which corresponds to the group of mice that experienced the strongest reduction in survival. The persistent increase in M2 macrophages in this group coincided with an increase in the immune suppressive mMDSC compartment at day 28 compared with the vehicle control mice (*p*_adj_ = 0.0228) (Figure 4e). Furthermore, the immune checkpoint molecule TIM-3 increased in the CaCO_3_ microparticle groups over time (Figure 4f). While at the early analysis time point the increase in TIM-3 concentrations was only present in the CaCO_3_-MP-A group compared with vehicle control (*p*_adj_ = 0.0017), at the latter analysis time point both CaCO_3_-MP-A and CaCO_3_-MP-C formulations elicited a significant increase in TIM-3 compared with vehicle control (*p*_adj_ = 0.0259 and *p*_adj_ = 0.0487, respectively). The increase in innate immune suppression was accompanied with an almost immediate suppression of the cytotoxic CD8 + T cells at day 14 in all mice that received CaCO_3_ microparticles compared with vehicle control mice (Figure 4g). However, statistical significance was reached for CaCO_3_-MP-A and CaCO_3_-MP-C (*p*_adj_ = 0.0071 and *p*_adj_=0.0034, respectively), while there was only a trend for the CaCO_3_-MP-D product formulation (*p*_adj_ = 0.0916). To summarize, most changes in immune status of the tumor were seen between the control and mice that received CaCO_3_-MP-A, in accordance with this group experiencing the strongest reduction in survival. Although there were trends of changes in some parameters, none were significant compared with the control group in mice receiving CaCO_3_-MP-D.

When it comes to differences between the CaCO_3_ microparticle formulations, significant changes were only found when CaCO_3_-MP-A and CaCO_3_-MP-D were compared. The fraction of M2 macrophages and concentration of TIM-3 at day 14 was significantly larger in mice who received the CaCO_3_-MP-A formulation compared with the CaCO_3_-MP-D formulation (*p*_adj_ = 0.0433 and *p*_adj_ = 0.0078), and similarly, on day 28 the fraction of mMDSCs was higher (*p*_adj_ = 0.0163). These differences were also reflected in the survival (Figure 4b). In contrast, no differences between CaCO_3_-MP-A and CaCO_3_-MP-C were observed, despite the statistically significant difference in survival.

### 3.4. The Dose of CaCO_3_-Microparticles Influences the Degree of Hyperproliferation

In this experiment, two different dosages (5 mg vs. 2 × 10 mg) of the same CaCO_3_ product formulation (CaCO_3_-MP-C) were evaluated (Figure 5b). Both groups of mice had a significantly shorter survival compared with vehicle control mice (median survival of 52 days), with the shortest survival in the highest dose group (median survival of 34 days, compared with 42 days for the 5 mg group with *p*_adj_ = 0.0062).

### 3.5. Chemotherapy Treatment Is Not Able to Sufficiently Reverse the Hyperproliferation Effect Seen with PLGA and CaCO_3_ Drug Carriers

Lastly, we assessed whether the hyperproliferative effect of IP injection of CaCO_3_ and PLGA particles in the ID8-fLuc ovarian cancer mouse model could be counteracted by administering chemotherapy one day after particle administration. When CaCO_3_-MP-B or PLGA were administered as a single treatment on day 13 post tumor cell inoculation, they both induced a hyperproliferation of the tumor as reflected by a reduced survival compared with vehicle control (median survival of 49 days, with *p*_adj_ = 0.0007, and 50.5 days, with *p*_adj_ = 0.0095, compared with 64 day, respectively). The addition of carboplatin and PLD was able to significantly prolong survival compared with single treatment with either CaCO_3_-MP-B (median survival of 82.5 days compared with 49 days, respectively, with *p*_adj_ = 0.0005) or PLGA (median survival of 91 days compared with 50.5 days, respectively, with *p*_adj_ = 0.0005). However, mice injected with CaCO_3_-MP-B or PLGA in combination with carboplatin and PLD showed a reduced survival compared with mice that were treated with carboplatin and PLD alone (median survival of 82.5 days, with *p*_adj_ = 0.0325, and 91 days, with *p*_adj_ = 0.0191, compared with 106 day, respectively). Unfortunately, chemotherapy treatment is only partially able to reverse this hyperprogression (Figure 6b), since it is clear that chemotherapy-treated mice still have a better survival than mice treated with chemotherapy combined with microparticles.

## 4. Discussion

In the development of new targeted therapies for ovarian cancer, an important focus is the use of drug carriers to specifically enhance drug retention at the tumor site—in this case, the peritoneal cavity. However, essential information concerning the effects of the individual drug carriers on tumor behavior and the TME is still lacking. In this study, we evaluated these effects after IP administration of different types of particle drug carriers in an immune-competent mouse model for ovarian cancer. Surprisingly, our results show that all particles included in the study can promote tumor growth and that the phenomenon has an immune etiology, as an increase in M2-like macrophages and MDSCs was detected. This indicates that the different particles elicit an immune response when injected IP and that the pro-tumoral effect seems to be mediated by the innate immune system through immunosuppressive mechanisms.

Previous findings published by our group suggest that innate immunosuppression dominates the adaptive immune response in the ID8-fLuc model, where MDSCs were identified as the key drivers of this immune suppression [29]. MDSCs also correlated with a worse prognosis in ovarian cancer patients at diagnosis [26]. Additionally, macrophage polarization towards an immune-suppressive and tumor-promoting M2 phenotype has previously been associated with ovarian cancer progression and malignant ascites development in preclinical [30,31] and clinical settings in humans [25,31,32,33]. In cancer patients, extensive progressive disease has been described after immune checkpoint blockade, with an incidence ranging between 4% and 29% for various tumor types [34,35]. At the moment, not much is known about the mechanisms behind such an outcome.

Concerning the direct effects of drug carriers on tumor development, the literature is scarce and provides contradicting information. While our study suggests a more favorable immune profile and somewhat better survival for pegylated liposomes compared with the other particle drug carriers, La-Beck et al. showed an increase in primary tumor growth and peritoneal metastasis driven by liposomes administered intravenously in the ID8 ovarian cancer model [36]. A similar hyperproliferation occurred after intravenous injection of liposomes in C57BL/6 mice bearing subcutaneous TC-1 tumors, a mouse model of human papilloma virus-induced cancer [37]. In contrast, intravenous injection of liposomes did not result in enhanced tumor growth in C57BL/6 mice bearing subcutaneously implanted B16-OVA melanoma cells [36]. Altogether, these results suggest that the immune-modulating effects of liposomes are probably more dependent on tumor characteristics than on features of the C57BL/6 host mice. In vitro, Lam et al. reported that the acid-neutralizing properties of CaCO_3_ nanoparticles can induce cancer cell reprogramming to suppress tumor growth and invasion in breast cancer cells [38], which is in contradiction to the hyperproliferation we have observed. It is clear from the divergent results in the literature that this is a complex topic. Most likely, many factors contribute and influence the outcomes, such as different product formulations of the drug carriers, the use of different tumor models, differences in administration routes and difference between in vitro vs. in vivo exposure.

That IP injections of particles can cause an immune response has also been confirmed in tumor-free naïve mice. Kohane et al. reported the presence of adhesions and chronic inflammation in response to PLGA microparticle administration in immunocompetent and tumor-free SV129 mice [39]. However, it must be noted that the mass dose in our studies (3–5 mg) was significantly lower than the mass doses in the study of Kohane et al. (10–100 mg). Additionally, Lebre et al. described an inflammatory reaction in immunocompetent and tumor-free C57BL/6 mice after hydroxyapatite administration [40]. Moreover, this response was largely dependent on the product formulation and size of the hydroxyapatite microparticles used, similar to the effects of different product formulations on tumor progression that were observed in the ID8-fLuc mouse model in the studies presented here.

An added survival benefit was not observed when we combined microparticle administration with chemotherapy treatment, in contrast with the studies mentioned in the introduction of this manuscript that obtained either an enhanced retention of the chemotherapeutic, prolonged survival, reduced tumor volume or complete tumor regression with chemotherapy-loaded microparticles [10,11,12,13]. Herein, the microparticles and the chemotherapeutics were administered as a co-treatment rather than microparticles loaded with a chemotherapeutic drug. Therefore, the advantage of enhanced drug retention within the peritoneal cavity is lost in our experiments, which may explain why an additional survival benefit was not seen in the ID8-fLuc mouse model compared with chemotherapy treatment alone. Additionally, we believe that the anti-tumoral effects of the chemotherapy regimen were not strong enough to fully counteract the pro-tumoral hyperproliferation effects caused by the microparticles in this case.

The effects from particles on tumor growth has several important implications for preclinical research on IP drug delivery systems for ovarian cancer. Since our results point towards the contribution of innate immune suppression to tumor progression, this highlights the need to use syngeneic mouse models in order to have a fully working immune system within the TME. If only human xenograft models in immunocompromised mice are used in the search for new drug delivery systems, potential immune modulatory effects of the carrier itself may be overlooked. Furthermore, aspects related to product formulation and dosing regimen also require thorough investigation. Although in the current study, hyperproliferation of the tumor is consistently present for different types of particle drug carriers (CaCO_3_, PLGA, hydroxyapatite and polystyrene) and in different product formulations of the same particle drug carrier (CaCO_3_), three important remarks have to be highlighted. First, hyperproliferation of the tumor could be minimized by reducing the mass dose of the drug carrier. For the particle drug carriers included in this study, a mass dose of 3 mg was able to eliminate the shortening of survival that is observed with higher doses of 5 or 10 mg. Second, the preparation of the product formulation of the drug carrier is important. For the CaCO_3_ drug carrier, not all product formulations caused the same degree of hyperproliferation. Moreover, while product formulations CaCO_3_-MP-A, CaCO_3_-MP-C and CaCO_3_-MP-D all caused a shortening of survival in the ID8 model when injected IP on day 1 post tumor cell inoculation, the optimized CaCO_3_-MP-B product formulation [18] did not show the same tumor hyperproliferation effect in terms of survival. This indicates the need to evaluate the product formulation of the individual drug carrier and optimize the production process before translating it to clinical applications. Third, the timing of injection is of importance. As stated above, the CaCO_3_-MP-B formulation did not result in a hyperproliferation of the tumor when administered on day 1 post tumor cell inoculation. However, when administered at a later disease stage (day 13), the CaCO_3_-MP-B did accelerate tumor progression that resulted in a reduced overall survival. This suggests that the tumor reacts differently to the microparticles depending on the tumor burden.

We would also like to acknowledge the fact that our study has some limitations. We recognize the lack of detailed information on the activation status and functional properties of the immune cells. However, the focus of our research was more to highlight the general shifts in immune cell populations. Furthermore, we acknowledge that the polarization of macrophages is a broad spectrum of which the pro-tumoral M2-like phenotype is just one extreme [41]. In our study, only CD206 was used as a specific marker for identifying the M2-like macrophage phenotype. We acknowledge the diversity and plasticity that exists with this cell type and that the classification in M1- and M2-like macrophages is an over-simplified phenotypic characterization. In future studies, a broader set of markers should be used to better identify this macrophage plasticity.

To which degree our findings are relevant in a broader scope remains to be seen. Our study focused only on the effects of particle drug carriers in the ID8 mouse model. In order to generalize our findings for ovarian cancer, the results need to be confirmed in other preclinical models. In addition, the current study only assessed whether chemotherapy treatment could reverse the tumor hyperproliferation caused by microparticles when added to the schedule as a separate treatment. To properly assess whether the pro-tumoral effects of the carrier itself can be counteracted, chemotherapy-loaded microparticles would have to be compared with standard chemotherapy treatment in order to take advantage of the prolonged IP residence time of the drug. This, along with systematic studies of the interactions between the immune system and drug carriers, can potentially contribute to an improved design of IP drug delivery systems in the future.

## 5. Conclusions

Intraperitoneal drug delivery by use of particle carriers is a potential new therapeutic option for ovarian cancer patients. Herein we provide essential information on the effects of several commonly investigated drug carriers on disease progression and TME in the ID8-fLuc mouse model, when the particles were administered without a drug payload. With this research we want to create awareness of the possible detrimental effects of drug carriers themselves, as we demonstrated that an IP injection of particles can result in hyperproliferation of the tumor, mediated by strong innate immune suppression. The results emphasize the need for using syngeneic tumor models when developing drug delivery systems and the importance of product formulation and dosing regimen, as reduction in the mass dose, changes in the formulation and adjustment of the timing of injection could diminish the negative effects. Therefore, we believe that these findings can contribute to future developments of novel and better targeted therapeutic strategies for ovarian cancer.

## Data Availability

The data are available from the authors upon request and with the permission of Oncoinvent AS, since the data are not publicly available.

## Supplementary Materials

The following supporting information can be downloaded at: https://www.mdpi.com/article/10.3390/pharmaceutics14040687/s1, Table S1: Antibodies used for T cell markers. Table S2: Antibodies used for myeloid markers.
