# Supplementary material for: Effect of Particle Carriers for Intraperitoneal Drug Delivery on the Course of Ovarian Cancer and Its Immune Microenvironment in a Mouse Model
Source: Pharmaceutics. 2022 Mar 22;14(4):687. doi: 10.3390/pharmaceutics14040687 (PMC9031420; doi:10.3390/pharmaceutics14040687)
Supplement: Supplementary file 1 [file pharmaceutics-14-00687-s001.zip › pharmaceutics-1629154-supplementary.pdf]

pharmaceutics

Supplementary Materials: Effect of Particle Carriers for Intra-peritoneal Drug Delivery on The Course of Ovarian Cancer and Its Immune Microenvironment in A Mouse Model

Roxanne Wouters, Sara Westrøm, Ann Vankerckhoven, Gitte Thirion, Jolien Ceusters, Sandra Claes, Dominique Schols, Tina B. Bønsdorff, Ignace Vergote and An Coosemans

Table S1. Antibodies used for T cell markers.

Target	Fluorophore	Supplier
FVD	eFluor 506	eBioscience
CD45	APC	eBioscience
CD3	APC eFluor780	eBioscience
CD4	PerCP Cy5.5	eBioscience
CD8	BV421	BD Bioscience
FoxP3	AF488	BD Bioscience

Table S2. Antibodies used for myeloid markers.

Target	Fluorophore	Supplier
FVD	eFluor 506	eBioscience
CD11b	PerCp Cy5.5	eBioscience
CD45	APC	eBioscience
Ly6G	Fitc	BD Bioscience
Ly6C	APC-efluor780	BD Bioscience
MHC II	PE-Cy7	eBioscience
F4/80	BV421	BD Bioscience
CD206	PE	Biolegend
